# Continuous monitoring of blood glucose using a fiberoptic-based intravascular sensor during postoperative care in the ICU

**DOI:** 10.1186/cc13632

**Published:** 2014-03-17

**Authors:** M Prasad, P Gopal, G Mannam

**Affiliations:** 1 Care Hospital Nampally, Hyderabad, India; 2 STAR Hospitals, Hyderabad, India

## Introduction

While there is ongoing discussion of the optimal range for glycemic control in hospital intensive care, recent publications from Mackenzie and colleagues [1] and Krinsley [2-4] suggest not only that mean BG should be considered, but also that glucose variability and complexity may be equally important. This has increased the need for continuous systems which can provide early warnings of hypoglycemia and effectively measure variability. GlySure Ltd has developed an intravascular glucose monitoring system to simplify the application of hospital protocols for tight glycemic control (TGC) at the point of care. Experience with the original research-based instrumentation [5] has now been incorporated into a combined pre-production monitor and autocalibration unit. We have now completed a 34-patient trial using this device and present the data collected from this study.

## Methods

The study used GlySure sterile, single-use sensors and a 5-lumen 9.5-Fr CVC device, allowing the fluorescence optical-based sensor to be placed into the patient's right internal jugular vein. The screen data were blinded to the bedside staff. Data from the monitor were later compared with sample measurement from the Yellow Springs (YSI) glucose analyzer. The data accuracy was measured using the mean absolute relative difference (MARD), an error calculation tool.

## Results

The device met the primary safety and effectiveness endpoints of the trial. The 456 sample values recorded by the monitor based on 8-hour calibrations were correlated with samples taken from the YSI and the MARD for the study was 9.40%. The analysis showed that 89.23% of the data fell within the A zone of the Clark error grid, with the rest falling within the B zone.

## Conclusion

The results demonstrate a good correlation with the accepted standard of blood glucose determination in ICU practice. Early detection of glycemic excursions can provide carers with the opportunity for an early intervention and thus achieve the elusive target of TGC around the chosen target range.
